# Courage as Mediator between Positive Resources and General/Domain-Specific Well-Being Indices

**DOI:** 10.3390/ejihpe12080076

**Published:** 2022-08-13

**Authors:** Ernesto Lodi, Lucrezia Perrella, Rita Zarbo, Patrizia Patrizi

**Affiliations:** 1 Department of Humanities and Social Sciences, University of Sassari, Via Roma, 151, 07100 Sassari, Italy; 2 Faculty of Human and Social Sciences, Kore University, Cittadella Universitaria, 94100 Enna, Italy

**Keywords:** courage, hope, optimism, flourishing, life satisfaction, academic satisfaction, college satisfaction, positive resources

## Abstract

Background: the current post-pandemic situation has exacerbated the effects already present due to the recent socio-economic crises belonging to the first two decades of this century: perception of instability, fears, concern for the future, underemployment, undignified work. This situation has negatively impacted life in general, career paths, and perceived quality of life, especially for new generations. Positive resources such as optimism and hope can have a positive effect in countering these effects which are impacting student academic satisfaction, life satisfaction, and flourishing. In the hypothesis, courage acts as a mediator for both domain-specific and general well-being, as perceived by college students. Methods: the research involved 410 Italian university students. Five rating scales were used: Visions About the Future, Courage Measure, Satisfaction with Life Scale (SWLS), the Flourishing Scale, and College Satisfaction Scale. Results: All research variables were significantly and positively correlated. The mediation model almost totally confirmed the hypotheses, as the relationship between optimism and academic satisfaction was totally mediated by courage. In other cases, optimism and hope had both a direct and an indirect effect, mediated by courage, on the life satisfaction, academic satisfaction, and flourishing of college students. Conclusions: the findings are discussed with recent theories and research on this topic, and suggestions are provided with practical implications for those involved in supporting the choice and construction of career paths.

## 1. Introduction

The current post-pandemic situation is characterized by insecurity, economic and social instability, unemployment, precariousness, and undignified jobs, and this is increasingly leading today’s young people to look to their future with fear and concern. These aspects can not only influence study and career paths, as well as the construction of careers themselves, but also the level of life satisfaction, quality of life, and well-being in general [1,2].

There is a nascent line of research that is turning to courage as a crucial factor in building career paths in contexts characterized by risks and fears, in addition to as a protective factor for university students’ well-being. Although the construct of courage has already been studied by many disciplines (even remotely in time, as the theme of courage was addressed by the ancient Greek philosophers starting from 400 BC), it has received only a little attention from the psychological disciplines, and many authors hope to increase empirical studies on this issue [2,3,4,5]. Courage, in fact, is considered a variable capable of supporting students in facing the risks and fears related to building careers and facing new challenges.

The main purpose of the study is, therefore, to provide further empirical evidence for the few studies that hypothesized courage as a mediating variable between psychosocial characteristics and various positive outcomes in the construction of careers and the well-being perceived in organizational and training contexts. We hypothesize that courage acts as a mediator between some positive resources of people (optimism and hope) and indices of general well-being and specific domains. While there are studies that have already focused on the mediator role of courage between positive resources (career adaptability, psychological capital, etc.) and general well-being indices, this study also has an innovative element in the evaluation of an index of specific well-being: academic satisfaction. In the construction of a career path, academic satisfaction can play a central role; it concerns the enjoyment of one’s academic experiences related to being a student [6] and the achievement of one’s academic goals and aspirations [7] and can influence the career choices and general well-being experienced by students. Indeed, multiple studies [7,8,9,10,11,12,13] have shown that academic satisfaction is positively correlated to many non-intellectual skills of students (self-efficacy, motivation, etc.), academic performance, and study paths coherent with students’ professional interests, while it is negatively correlated with professional indecision [8,9,10,11,12,13].

Two theoretical approaches have influenced most of the studies that considered the role of courage in career building and promoting well-being levels in organizational and educational contexts: the life design paradigm and positive psychology. These are the fundamental theoretical approaches for the present study as well.

The life design paradigm looks at career development as the result of a dynamic interaction between the individual and the context. This paradigm emphasizes the need to develop resources to support people in planning their future, despite uncertainties or disadvantages, so they can face hostile working conditions, adapt to changes, and, in general, experience increased well-being [14]. In the current socio-economic context, particular importance is attributed to courage, and it is considered a central variable for the construction of career paths. In fact, courage is also fundamental for promoting well-being during the career-building process and when facing any challenges, risks, and fears and possible inconveniences due to the above situation, increasing perseverance in achieving significant goals [3,15,16].

In terms of well-being, the positive psychology approach has assumed a predominant role over the past two decades, shifting the focus from people’s gaps and dysfunctions to their resources and strengths. Starting from its principles, from the perspective of prevention and promotion of health, well-being, and quality of life [17], it is central to enhancing individual and contextual resources. Positive psychology promotes the skills, competencies, and attitudes required to face the complex reality in which new generations live: a risk society experiencing globalization, social insecurity, precariousness, and instability [18,19,20]. In this context, variables, such as optimism, which is understood as the ability to look positively to the future and the tendency to attribute to oneself the ability to react to stressful situations, and hope, which is understood as the ability to set goals, identify the strategies necessary, and the ability to self-motivate to achieve them, could be key factors for well-being and career paths. Courage also takes on special significance in positive psychology. In fact, Peterson and Seligman [21] highlighted that courage represents one of the six dimensions of human strength and virtue, helping people to achieve optimal functioning and to flourish.

Given these premises, it is now more important than ever for career counselors and all professionals who deal with career choice support to commit to supporting successful career paths for the person to increase their chances of experiencing high levels of well-being in every context on their educational path, at all levels, and in their work [8,22]. The importance of optimal psychological functioning has been emphasized as an essential component in the assessment of well-being [23,24]. Flourishing is the perception that a person has of his being able to fully realize his potential and qualities, not only related to individual self-realization but also to positive functioning in his relationship with the world [25,26].

In this scenario, positive variables such as hope, optimism, and courage can foster higher levels of academic satisfaction, life satisfaction, and flourishing and, more generally, can help university students to cope better and more effectively with situations of uncertainty by increasing levels of perceived quality of life.

## 2. Literature Review

In this analysis of the theoretical background, the single theoretical constructs (optimism, hope, courage), their definitions, and their reciprocal relationships are deepened. In particular, the link between the previously mentioned constructs, both in relation to career paths (from the perspective of life design) and in relation to the levels of quality of life experienced by people and the relationship between life satisfaction, academic satisfaction, and flourishing, and, therefore, also the connection between these constructs and the theoretical principles of positive psychology, is investigated.

### 2.1. Optimism

Optimism is defined as the propensity to believe that positive future life scenarios can be built and achieved, attributing to oneself, above all, the ability to do so. It refers to the way in which people explain the causes and implications of events in terms of internality, stability, and globality. In this sense, an optimistic person tends to consider success as a result of his actions [27].

Optimism is characterized by the ability to look at life to take full advantage of experience, one’s abilities, and the opportunities offered to the benefit of one’s future. It is the propensity to find the best consequences even from events over which no influence can be exerted [28]. Optimism is related to having a better level of physical health, quality of life, psychological well-being, hope, resilience, self-esteem, and social skills [29,30,31,32] and is related to a wide range of well-being indicators: lower levels of anxiety and depression, fewer suicide attempts, fewer maladaptive behaviors, more positive perceptions of oneself and the future, less chance of being victimized or excluded by others, higher sociometric indices, better social relationships, a more positive perception by others, and a greater ability to react to stressful events [29,30,31,32,33,34,35]. Optimism, therefore, seems to be associated with more positive perceptions about the future [36,37,38,39] and a greater ability to react to stressful situations and, therefore, is a predictor of psychological adaptation.

Optimism has positive correlations with positive indices of subjective well-being (SWB), such as life satisfaction, understood as a cognitive process based on which the person expresses an awareness and qualitative judgment about his life in relation to a series of personally established criteria [40,41]. Indeed, optimism is an important predictor of life satisfaction [34,36,42,43,44,45,46], as optimistic people rate their satisfaction with life more positively than pessimists, regardless of age and even after adapting to the stage of life they are in. The same is also true for undergraduate students [34,47,48,49,50,51,52,53,54], also highlighting that the negative effect of stressful events experienced in academic settings on life satisfaction does not affect students with high levels of optimism; this is considered fundamental for the life satisfaction and psychological well-being of university students.

There are currently only a few studies that have specifically investigated the relationship between optimism and academic satisfaction. Some studies highlighted that optimism is associated with higher academic satisfaction than pessimism [47,55,56]. In particular, the study by Boileau et al. [47] highlighted that task-oriented coping represents a significant mediator of this association and that a student’s daily satisfaction can vary depending on the coping strategies used by that person at that time.

Although there are not enough studies on the relationship between optimism and flourishing and, therefore, there is a need for further study, optimism seems to also be related to flourishing [57,58,59,60]. According to the study by Finch et al. [57], optimism, respect for hope, self-efficacy, and resilience are the strongest predictors of flourishing [54].

Overall, students with higher levels of optimism appear both to have greater resources for career building, for example, greater positive visions about the future and the coping skills and ability to cope with stressful events, and to experience more academic satisfaction in their life in general and experience more flourishing.

### 2.2. Hope

Snyder and colleagues [61,62] defined hope as the motivation that is nurtured towards the possibility of achieving certain results and objectives. Having hope for your future means believing in your abilities and aiming for improvement. Hope allows people to gather energies and use them to achieve dreams and goals while maintaining commitment and the desire to reach those goals.

Hope is articulated as the ability to: (a) clearly focus on goals, (b) identify specific strategies to achieve these goals, and (c) stimulate and support the motivation to use these strategies [61,62]. Hope has positive correlations with many subjective well-being indicators (SWB); for example, hope represents an important predictor, both direct and indirect, in determining people’s levels of life satisfaction [34,63,64,65,66,67,68,69]. In particular, the study by Yaghoobzadeh et al. [69] highlighted how a person’s ability to be hopeful positively predicts life satisfaction. In this sense, hope supports the person to initiate useful actions to achieve goals and overcome any difficulties and/or obstacles, thus representing an important motivational factor that supports people to resist the adversities of life, to feel more capable of facing stressful events, and to perceive greater well-being. Furthermore, some studies also underlined the important, protective role that hope and satisfaction with life play concerning the development of depressive symptoms, health risk behaviors (use/abuse of drugs), stress, and lower quality levels of life [63,70,71].

Hope is also a predictor of academic satisfaction. Some studies showed that higher levels of hope in students are positively correlated with academic satisfaction [72,73]. Students with high academic satisfaction levels show significantly higher levels of life expectancy and satisfaction [73]. However, there are not enough studies regarding academic satisfaction, and only some of them focused on job satisfaction, highlighting the positive association between hope and job satisfaction [74,75,76,77].

Hope is an important predictor of flourishing as well. In particular, the literature emphasized not only the positive correlation between hope and flourishing [26,78,79,80,81,82,83,84] but also between the components of hope (agency and pathways) and flourishing [78,79,85]; agency and pathways represent fundamental, if not crucial, variables for experiencing positive and optimal psychosocial functioning.

As also clearly highlighted by the study by Yaghoobzadeh et al. [69], hope is linked both to central variables for career development (i.e., achievement of objectives, overcoming of difficulties, ability to resist difficulties) and to people’s levels of well-being.

### 2.3. Courage

Within the theoretical framework of positive psychology, courage is described as an important virtue [21,86]. It can be defined as the ability to act despite fear [87], intentionally and for a noble purpose [88].

A few pieces of research in recent years have investigated the role that courage can play in different life contexts and in relation to indicators of psychosocial adaptation, well-being, and quality of life in various age groups.

For example, the tendency of some individuals to act courageously from infancy, as well as the significant positive relationship between courage and extroversion and negative relationship between courage and anxiety in childhood, has been demonstrated [89,90,91].

It has also been shown that, even in adolescence, young people with higher levels of courage, being able to manage the feeling of fear and possessing greater coping skills for dealing with situations, are likely to be more motivated to achieve their goals by devising and deploying alternative solutions [4,92].

Moreover, in a sample of 500 people aged between 18 and 60, Magnano et al. [4] observed that courage can be considered a protective factor in stressful conditions, and it is positively related to the ability to cope with stressful situations and negatively related to avoidance. Other studies showed positive correlations between character strengths, including courage, and subjective physical and mental well-being [93,94].

Courage is also predictive of a lower occurrence of psychological symptoms such as paranoid ideation and obsessive-compulsive symptoms [95]; it positively contributes to life satisfaction [96], and it helps people to resist external problems and stimulates agency [97,98,99]. Courage has also been shown to play an important role in the achievement of goals related to the work context [100,101] as well as in better academic performance [102]. Moreover, research that focused on dimensions related to psychological capital, including hope and optimism, showed the positive impact these resources have on flourishing, both directly and indirectly, through the mediation of courage [3]. The close relationship between courage, optimism, and hope was also underlined by the suggestions of some scholars who included courage as an additional dimension among the other dimensions that constitute psychological capital (hope, self-efficacy, resilience, and optimism) [96,103]. Furthermore, a study conducted by Hannah and colleagues [104] found that courageous behavior can be stimulated by other positive personal dimensions and resources such as resilience, optimism, hope, and openness to experience. These dimensions act in reducing feelings of fear by increasing people’s likelihood to engage in courageous behavior despite perceived risk and improving quality of life. Finally, recent studies, conducted during the COVID-19 pandemic, showed that personal positive resources (i.e., optimism, hope, courage, trait mindfulness, and self-efficacy) can directly influence resilience, which, in turn, prevents psychological distress [105].

In the analysis of this construct, a link between courage and a positive attitude toward risks and fears associated with the construction of career paths, as well as the contribution of courage as a mediating variable in favoring the growth of well-being in both the general and specific domain of people, emerged.

## 3. Aims of the Study and Hypothesis

Given the scenario set out above, courage can mediate the effect of hope and optimism on specific and general student well-being, precisely because they are living in a period of great uncertainty. Having high levels of hope and optimism has already been shown to affect perceived levels of well-being, but it is believed that the propensity to act courageously could facilitate a positive level of academic satisfaction, life satisfaction, and flourishing. In fact, even having high levels of courage could help students to face uncertain situations, promoting the level of perceived quality of life more effectively. Thus, the hypothesis is that the dimensions of positive resources, such as optimism and hope, can have both a direct and indirect effect on students’ academic satisfaction, life satisfaction, and flourishing; the indirect effects are hypothesized to be mediated by courage. Specifically, the hypotheses are the following:

**Hypothesis** **1** **(H1).** *Hope has both a direct and indirect effect, mediated by courage, on academic satisfaction (H1a), life satisfaction (H1b), and flourishing (H1c).*

**Hypothesis** **2** **(H2).** *Optimism has both a direct and indirect effect, mediated by courage, on academic satisfaction (H2a), life satisfaction (H2b), and flourishing (H2c).*

## 4. Method

### 4.1. Participants

Participants were 410 Italian university students aged between 18 and 30 years (mean age = 23.70; S.D. = 4.40) not balanced by gender (80% F, 20% M). Of the participants, 66.9% were completing a three-year degree course (first level), 19% a two-year degree course (second level), and 14.1% a master’s degree course (single cycle of 5 years). The degree courses fell in the following areas: human and education sciences (29.2%); biomedical science (21.3%); architecture, cultural heritage, and disciplines of art and entertainment (10.5%); letters, history, and languages (12.7%); law and political and economic sciences (14.7%); engineering and computer sciences (7.2%); and communication sciences (4.3%). Of the sample, 16.7% carried out a job at the same time as studying.

### 4.2. Measures

**Vision About Future** [106] evaluates 22 items on a 5-point Likert response scale: (a) Optimism (7 items; an example of item: “Generally I am full of enthusiasm and optimism”; alpha: 0.84; alpha study sample: 0.91); (b) Negative vision of the future (6 items, example of item: “I often feel that things will go wrong”; alpha: 0.81); and (c) Hope (5 items, an example of item: “In the future, I will be involved in very important projects”; Cronbach’s alpha: 0.84; Cronbach’s alpha study sample: 0.91). In this study, only the 2 sub-dimensions that measure optimism and hope were used.

**Courage Measure:** the Italian version of the Courage Measure (CM) [87,107], adapted from the short version by Howard and Alipour [108], consists of 6 items (e.g., “I tend to face my fears”) structured on a seven-point Likert response scale which ranges from 1 (never) to 7 (always). In the sample, the measure showed a good level of internal consistency (Cronbach’s alpha = 0.88.).

**Satisfaction with Life Scale (SWLS)** [109]: the Italian version of the SWLS [110] evaluates overall life satisfaction through 5 items on a 7-point Likert scale (1 = strongly disagree; 7 = strongly agree; sample item: “In most ways, my life is close to my ideal”). The Cronbach’s alpha of the validation study was 0.88; in this study sample it was 0.85. The SWLS is the most-used scale for subjective well-being, and, recently, the scale has demonstrated measurement invariance across 24 countries, age, and gender [111].

**The Flourishing Scale** [112]: the Italian version, validated by Di Fabio [113], measures meaning and purpose in life using a one-dimensional approach (sample item: “I am engaged and interested in my daily activities”). The scale is composed of 8 items on a 7-point Likert scale (1 = strongly disagree; 7 = strongly agree). The Cronbach’s alpha in the validation study was 0.88, and, in our study, the sample was 0.84.

**College Satisfaction Scale (C-Sat Scale; [114]):** this instrument evaluates, using 20 items rated on a Likert scale (from 1 “not at all satisfied” to 5 “completely satisfied”), the degree of college satisfaction in a multidimensional way with five sub-scales related to different aspects of the college experience. It assesses the following areas: Choice (appropriateness of the student’s college choice), Services (quality of the university’s services), Relationships (quality of relationship with colleagues), Study (quality of study habits), and Usefulness for a Future Career (perceived utility of course attended for the career path). In the first validation study [114], all scales showed good reliability with McDonald’s omega indices from 0.80 to 0.92 and good CFA fit indices. A global measure of academic satisfaction was used for this study (Cronbach’s alpha = 0.92).

### 4.3. Procedure

Students gave their consent to participate in the research and, aware of the future publication of the general results, they completed an online survey. The online survey was built by transposing the standardized scales and questions about demographic data onto a Google form.

Participants were recruited according to a criterion of convenience: (a) by placing advertisements on the institutional websites of the two universities involved in the research; (b) using social platforms; (c) by disseminating information about the possibility of taking part in research during the lessons of university courses through the involvement of other teaching colleagues from both universities. Furthermore, no exclusion criteria were applied.

The questionnaire, which was filled in individually, took approximately 15–20 min to complete. Participants were free to leave the study at any time. To respect anonymity and to avoid double compilation, each student was asked to assign themselves a code using the two initial letters of their name and surname and their date of birth.

The ethical committee of the University of Sassari approved the research (project no. 2022-UNSSCLE-0061755), and it followed the rules of the Italian Association of Psychology for psychological research and the Helsinki Declaration.

### 4.4. Data Analysis

The software used for the data analysis was: (a) IBM SPSS Statistics, version 25.0. (Armonk, NY, USA: IBM Corp.) to calculate descriptive statistics and correlations; and (b) JAMOVI 2.2.5 [115] to test the mediational hypotheses and to conduct the path analysis. Preliminary analysis was conducted to check missing values, normality of distribution, and socio-demographic differences in the variables of interest of the study (no differences emerged).

## 5. Results

In Table 1, descriptive statistics and correlations between all the variables are reported. All items and scales showed a normal distribution (skewness and kurtosis from −1 to 1). All the variables were significantly and positively correlated.

### Mediation Analysis

In this study, the significance of indirect effects is reported using the bootstrap method (5000 repetitions, 95% confidence interval (CI), which indicates the significance of the effect with a probability of error of 5% (the CIs that do not include 0 are significant)), and the β is standardized to indicate the intensity of the effect.

**Figure 1:**
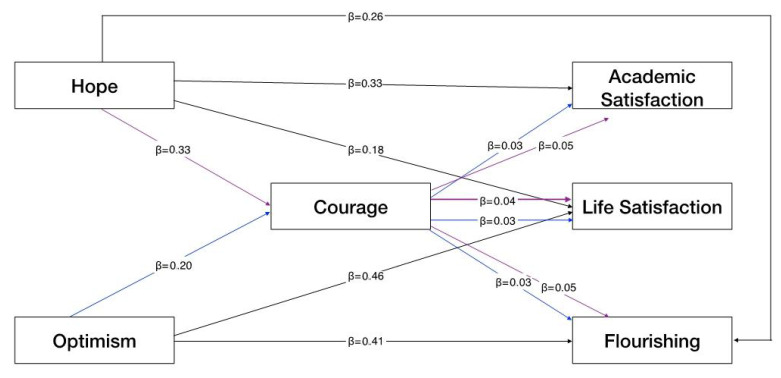
The mediation model between positive resource and well-being indices with courage as mediator.

Hope and optimism had both a direct effect (DE) and an indirect effect (IE), mediated by courage, on life satisfaction, academic satisfaction, and flourishing. The only path that was not statistically significant (but still at the limit: *p* = 0.080) was the direct relationship between optimism and academic satisfaction; this relationship was fully mediated by courage. Figure 1 presents the mediation model.

Specifically (Table 2), the results showed that hope had a direct relationship with life satisfaction (DE = 0.175, CI = 0.069–0.296), academic satisfaction (DE = 0.332, CI = 0.491–1.060), and flourishing (DE = 0.260, CI = 0.208–0.470); moreover, the paths from courage to life satisfaction (IE = 0.044; CI = 0.009–0.079), academic satisfaction (IE = 0.047; CI = 0.020–0.200), and flourishing (IE = 0.049; CI = 0.021–0.105) were significant, showing a direct and indirect effect of hope on all the three well-being indices mediated by courage.

Optimism had a direct relationship with life satisfaction (DE = 0.460, CI = 0.4466–0.698) and flourishing (DE = 0.411, CI = 0.485–0.800) but not with academic satisfaction; moreover, the paths from courage to life satisfaction (IE = 0.026; CI = 0.006–0.064), academic satisfaction (IE = 0.028; CI = 0.015–0.159), and flourishing (IE = 0.030; CI = 0.006–0.087) were significant, showing a direct effect of optimism on life satisfaction and flourishing and an indirect effect on all the three well-being indices mediated by courage.

The path analysis, conducted to estimate the relationships between the links and to confirm the information on the causal processes underlying the model relating to exogenous and endogenous variables, showed a model with good fit indices: χ^2^/df = 3.66; CFI = 0.99; RMSEA = 0.08; SRMR= 0.02. Therefore, the chi-square/df ratio was between 3 and 5, and the CFI was between 0.95 and 1; an acceptable RMSEA is <0.09, an acceptable SRMR is <0.08 [116,117,118].

## 6. Discussion

The aim of the study was to analyze the role of courage as a mediating variable between some positive resources of people (optimism and hope) and indices of general well-being and specific domains. In particular, the study sought to investigate the relationship between hope and optimism in life satisfaction, academic satisfaction, and flourishing. It was hypothesized that hope (hypothesis 1) and optimism (hypothesis 2) have both a direct and indirect effect, mediated by courage, on academic satisfaction, life satisfaction, and flourishing. The results mostly confirmed the hypothesis of the study: optimism and hope have both a direct and indirect effect on students’ academic satisfaction, life satisfaction, and flourishing, and the indirect effects are mediated by courage.

More specifically, we can now discuss the single patterns that emerged in our model related to the two main hypotheses.

The results confirmed that hope has both a direct and indirect effect, mediated by courage, on academic satisfaction (H1a), life satisfaction (H1b), and flourishing (H1c). These results confirm what emerged from the analysis of the literature; people who have hopeful visions, and are, therefore, able to set goals imagine alternative strategies to achieve them, and who show the ability to motivate themselves are probably also those who experience higher levels of life satisfaction [69], flourishing [72,73], and domain-specific well-being [78,79,85]. The significant, mediating role of courage suggests that hope is positively linked to self-perceived courageousness, which, in turn, is positively linked to all the indices of well-being. Students with higher levels of hope may perceive themselves to be more able to act courageously in pursuing their future aims despite perceived risks and fears, and this may promote a more positive judgment of their life, their student experience, and their perception of and ability to use their full potential. Hope, in fact, supports the person to initiate useful actions to achieve goals and overcome obstacles, especially in their career path, as Yaghoobzadeh and colleagues [69] underlined. Finally, our study expands the literature on the link between hope and academic satisfaction, since, as reported above, there are only a few studies present in this field.

Optimism has both a direct and indirect effect, mediated by courage, on life satisfaction (H2b) and flourishing (H2c), while the path from optimism to academic satisfaction is fully mediated by courage (H2a). These results confirm what emerged from the analysis of the theoretical background; people who have a higher level of optimism, stronger beliefs that positive future life scenarios can be built and achieved, and a higher propensity to consider success as a result of their actions are probably also those who experience higher levels of life satisfaction [42,46] and flourishing [57,58,59,60]. The significant, mediating role of courage suggests that optimism is positively related to self-perceived courageousness, which, in turn, is positively linked to the general indices of well-being. Students with higher levels of optimism may perceive themselves to be more able to act courageously in pursuing their future aims despite perceived risks and fears, and this may promote a more positive judgment of their life and their perception of and ability to use their full potential. In fact, optimism is related to a greater tendency to react to stressful situations, and it is a predictor of psychological adaptation. Therefore, as Hannah and colleagues [104] stated, courageous behavior can be stimulated by other positive personal dimensions and resources such as optimism.

As said before, there are only a few studies that have investigated the relationship between optimism and academic satisfaction. Contrary to expectations, although we have a few studies that confirmed that optimism is more associated with higher academic satisfaction than pessimism [47,55,56], hypothesis 2a was only partially confirmed, as optimism did not show a significant direct effect (even within the limits of significance) on academic satisfaction but a totally courage-mediated effect. We believe that more extensive studies are needed on this to deepen the link between optimism and academic satisfaction and the mediating role of courage. The explanation that we can still try to give is that the current pandemic situation, the effects of which are still evident today, has probably led people to perceive that they have less control over events (and so experience a diminution of explanation and implications of events in terms of internality and stability), especially over the ones related to educational, training, and work contexts. In this sense, the recent pandemic situation could have affected the relationship between optimism and academic satisfaction. This did not happen with hope in this relationship (it had a significant direct effect on academic satisfaction) because, if we focus on the definition of hope, it is more related to the concept of aims than optimism (a more general dimension), and, for this reason, is closer to the domain-specific context of the university. Finally, our study expands the literature on the link between optimism and flourishing, as we do not have enough studies in this field.

In general, we can affirm that our results are in line with what was theorized by the life design paradigm and positive psychology; students have adopted more optimistic and hopeful visions of the future, with the contribution of courage, that can help them in their perception of being able to counter fear and act proactively in the face of adverse and stressful circumstances, despite concerns, and they appear to have greater well-being. In this sense, students with higher levels of optimism and hope may have greater resources for building career paths since recent literature showed the strong link between well-being and positive outcome in careers (achievement, motivation, etc.). Students with a higher level of optimism, hope, and courage can maybe show a greater propensity for achieving goals, overcoming difficulties, and experiencing greater well-being, specific domain satisfaction, and a more thriving life experience.

The results of this study also seem to increase knowledge about the construct of courage, especially concerning the role it can play in supporting university students in facing the risks and fears related to building careers and new challenges and in mediating the effects of positive variables such as optimism and hope on the indices of general well-being and specific domains.

The results of the study led to some reflections on the practical implications. The current scenario presents professionals, counselors, and professors with numerous challenges since, today, they are called to promote more and more actions aimed at facilitating and supporting university students’ lives and career trajectories. It is necessary to rethink the construction of services, courses, and training to support students with regard to a different perspective of growth and “resilient” development and to improve the quality of life of university students and their well-being. This means knowing how to support university students through actions that aim to help them to know how to listen and build their aspirations, as well as enhance their strengths as central aspects to effectively counter and manage any accidents along the way; today, these accidents are more likely possible due to the increase in social and economic precariousness and instability in general. In this sense, for example, in university counseling services, it is important to dedicate counseling sessions, both individual and group, to specific training on courage, hope, and optimism, as the promotion of these resources is useful, for example, to students for countering concerns, fears, uncertainties, and obstacles for their future and allows them to feel capable of performing resilient behaviors when planning actions to face any difficulties. The results of the study, in fact, underline the importance of proposing paths that promote the subjective well-being of students to facilitate their study and career path, especially in a difficult historical moment with uncertainty as to what they are currently experiencing [3,4,34,42,66,74,91,92,96]. In this scenario, the need for specific training to train professionals in the themes of hope, optimism, and courage and on how to enhance these variables in professional practice with students, has also emerged as an indispensable tool for increasing students’ lives and career well-being, as demonstrated by the research in this field.

### Limitations and Future Research

The results of this study should be read with due attention to avoid possible generalizations of the results as they have some limitations. In fact, a sample of convenience was used from only two Italian universities, moreover, only from the two major islands: Sardinia and Sicily. For this reason, cultural influence and bias may have influenced the results since the condition of the regions may be different at many levels from other regions and countries. Furthermore, the variables of gender and the different courses followed were not balanced; this could have led to further distortions concerning external variables not considered in the mediation model. In addition, the following methods were used: (a) self-reporting measures, which could have influenced the validity of the study in terms of data reliability; and (b) a cross-sectional research design that did not allow for fully validated hypotheses of a causal nature. For this reason, longitudinal studies will need to be conducted in future research on this topic.

Future research is needed to better clarify the role of courage as a mediator between positive resources (not only hope and optimism but also, for example, resilience and other virtues/strengths) and to confirm the impact on different indices of general and specific well-being of the domain. Future research could be conducted using or creating ad hoc specific training on hope, optimism, and courage to verify, over time, whether these variables can promote the general and domain-specific well-being of students, supporting them not only in career planning but also in experiencing higher levels of quality of life in university contexts and life in general. In addition, further future research on the effectiveness and efficiency of methods/training that could better support professionals in working with students concerning the acquisition and/or development of the positive resources analyzed in this study would be appropriate. In fact, professionals and career counselors need specific and high-quality training on how to use specific career counseling sessions on hope, optimism, and courage to improve the well-being of students.

## 7. Conclusions

The current socio-economic context, aggravated by the pandemic situation, the effects of which are still evident today, has led people to perceive that they have less control over events, especially the ones related to educational, training, and work contexts. That period has amplified people’s experiences of precariousness and instability, putting a strain on the capacity of resistance, restructuring, and coexistence with emotions and moods, sometimes generating forms of malaise which are considered real barriers to the realization of life and career paths.

In this scenario, and as our research seems to confirm, positive variables such as optimism, hope, and courage can foster higher levels of academic satisfaction, life satisfaction, and flourishing and, more generally, can help university students to cope better and more effectively with situations of uncertainty by increasing the levels of quality of life. In fact, optimism and hope play a central role in some of the major indicators of psychosocial adaptation and quality of life and in determining the well-being levels of university students. Courage, and, consequently, courageous behavior, makes it easier for students to be ready to face risks, precariousness, uncertainty, and instability. These aspects are of crucial importance for supporting the regularity of the students’ study paths and their well-being while studying and building career paths. Courage, hope, and optimism can make it easier for university students to overcome adversity and promote well-being in a complex historical period where unpredictable changes can also be read as opportunities (not just limits) for development and growth in life and career paths.

## Figures and Tables

**Table 1:** Descriptive statistics and correlations between the variables (Pearson’s r).

	M	SD	1	2	3	4	5	6
1. Hope	24.45	6.00	1					
2. Optimism	14.31	4.94	0.68 ***	1				
3. Courage	26.84	7.71	0.47 ***	0.42 ***	1			
4. Academic satisfaction	70.22	14.03	0.48 ***	0.40 ***	0.35 ***	1		
5. Life satisfaction	19.67	6.16	0.55 ***	0.64 ***	0.41 ***	0.55 ***	1	
6. Flourishing	39.57	7.75	0.61 ***	0.65 ***	0.45 ***	0.57 ***	0.70 ^***^	1

*** *p* < 0.001.

**Table 2:** Effects of hope and optimism on well-being indices through courage (standardized βs).

Paths	Indirect Effect (IE)			Direct Effect (DE)			Total Effect		
	β	C.I. 95%		β	C.I. 95%		β	C.I. 95%	
Hope–Courage–Life satisfaction	0.044	0.009	0.079	0.175	0.069	0.296	0.219	0.121	0.328
Optimism–Courage–Life satisfaction	0.026	0.006	0.064	0.460	0.446	0.698	0.486	0.481	0.732
Hope–Courage–Academic satisfaction	0.047	0.020	0.200	0.332	0.491	1.060	0.379	0.613	1.157
Optimism–Courage–Academic satisfaction	0.028	0.015	0.159	0.114	-0.037	0.687	0.142	0.073	0.734
Hope–Courage–Flourishing	0.049	0.021	0.105	0.260	0.208	0.470	0.310	0.274	0.523
Optimism–Courage–Flourishing	0.030	0.006	0.087	0.411	0.485	0.800	0.440	0.540	0.842

## Data Availability

The data presented in this study are available on request from the corresponding author.
